# Proteomic response in *Streptococcus gordonii* DL1 biofilm cells during attachment to salivary MUC5B

**DOI:** 10.1080/20002297.2021.1967636

**Published:** 2021-08-23

**Authors:** Carolina Robertsson, Gunnel Svensäter, Zoltan Blum, Magnus E Jakobsson, Claes Wickström

**Affiliations:** a Department of Oral Biology and Pathology, Faculty of Odontology, Malmö University, Malmö, Sweden; b Department of Biomedical Science, Faculty of Health and Society, Malmö University, Malmö, Sweden; c Department of Immunotechnology, Lund University, Lund, Sweden

**Keywords:** Salivary mucin, MUC5B, oral streptococci, *Streptococcus gordonii*, oral biofilm, saliva, mass spectrometry, protein expression, proteomics

## Abstract

**Background:**

Salivary mucin MUC5B seems to promote biodiversity in dental biofilms, and thereby oral health, for example, by inducing synergistic ‘mucolytic’ activities in a variety of microbial species that need to cooperate for the release of nutrients from the complex glycoprotein. Knowledge of how early colonizers interact with host salivary proteins is integral to better understand the maturation of putatively harmful oral biofilms and could provide key insights into biofilm physiology.

**Methods:**

The early oral colonizer *Streptococcus gordonii* DL1 was grown planktonically and in biofilm flow cell systems with uncoated, MUC5B or low-density salivary protein (LDP) coated surfaces. Bacterial cell proteins were extracted and analyzed using a quantitative mass spectrometry-based workflow, and differentially expressed proteins were identified.

**Results and conclusions:**

Overall, the proteomic profiles of *S. gordonii* DL1 were similar across conditions. Six novel biofilm cell proteins and three planktonic proteins absent in all biofilm cultures were identified. These differences may provide insights into mechanisms for adaptation to biofilm growth in this species. Salivary MUC5B also elicited specific responses in the biofilm cell proteome. These regulations may represent mechanisms by which this mucin could promote colonization of the commensal *S. gordonii* in oral biofilms.

## Introduction

The mucous layer on the epithelia of the gastro-intestinal tract (GIT), largely made up of mucins, acts as a first line of defense against colonizing pathogens and commensals [1]. As a part of the innate immunity, the viscous coat protects underlying tissues against microbial challenges by forming a physicochemical barrier; nonetheless, numerous specific bacterium-mucin-interactions also occur. Many species can colonize the mucous layer, and by providing adhesion sites, nutrients and other chemical cues for bacterial activity and virulence, mucins or products from mucin degradation modify the attachment, diversity, and activity of bacterial biofilms in the GIT. For a review on interactions between intestinal bacteria and mucins that coat the GIT, see Sicard et al. [1]. Modification of local environments and selectivity through specific interactions are major determinants for biofilm development [2,3]. The activities of early colonizers, such as *Streptococcus gordonii*, modulate plaque maturation and may enable later colonization of more dysbiosis-related species, such as the caries-related streptococcus *Streptococcus mutans* and others [4–6]. Even though the oral cavity is indeed a part of the GIT, specific interactions between salivary mucins and oral biofilms are not well studied.

Salivary mucin MUC5B, also found in the pellicle that forms on all oral surfaces, is a structurally important component of saliva [7]. MUC5B, similarly to mucins on mucosal surfaces elsewhere in the body [1], displays dual functions in microbial adhesion by providing nutrition and attachment sites that promote colonization by oral bacteria while also modulating the biofilm through mobilization of specific cellular activities, and is thus suggested to affect biofilm activity and succession [8,9]. The MUC5B molecule consists of numerous subunits with repeated mucin domains of highly glycosylated polypeptide segments [10]. This constitutional complexity seems to promote biodiversity in dental biofilms by promoting synergistic ‘mucolytic’ activities in a variety of microbial species that need to cooperate to release nutrient components from all sections of the complex glycoprotein [8,11,12].

It is recognized that biofilm bacteria behave differently compared to planktonic cultures [13]. Differences in intracellular protein expression in planktonic and biofilm phenotypes have been demonstrated in many bacteria [14,15], including oral streptococci [16]. However, few studies examine changes in the cellular proteome of oral biofilm bacteria induced by attachment to isolated surface-bound components of human saliva. Previous work from our laboratory investigated the cell surface proteome of *S. gordonii* DL1 [17], and established that adhesion to MUC5B induced the expression of a novel cell surface protein in biofilm cells of this species (unpublished data). The current study aims to identify events that occur inside the cell as a consequence of outside-in signaling in biofilm cultures during attachment to this mucin. Salivary MUC5B was isolated by methods designed to retain its native polymeric structure and physiological integrity [18], to attempt to better reproduce *in vivo* interactions between oral biofilm bacteria and flow cell surfaces coated with this salivary mucin.

Contesting the old standard of static modelling, efforts have been made to develop and improve flow cell models for studying biofilm physiology [19]. Mimicking *in vivo* conditions using flow cell models, however, presents several challenges [20]. Biofilm architecture is affected by the character of the flow, for example, compared to laminar flow, turbulent flow produces more filamentous structures in the biofilms [21] and intense flow forces may reduce the volume of biofilms by physically removing clusters of cells [22]. Elimination of artefacts caused by accumulation and sedimentation of material are however important benefits of employing flow cell modelling in the study of biofilms, together with the simultaneous selection of cell populations with increased biofilm formation capacities. In the current study, a laminar flow cell system with low shear forces used in previous experiments with oral streptococcal biofilms [23] was employed.

To explore bacterial adaptation to the biofilm lifestyle, and specific responses elicited during attachment to salivary MUC5B, the cellular proteomes of *S. gordonii* DL1 planktonic cultures and flow cell biofilms grown on uncoated or salivary MUC5B or low-density salivary protein (LDP) pre-coated surfaces were investigated. Changes in the proteomic profiles of oral commensals in response to attachment to MUC5B are possibly associated with regulation of events involved in sustaining biosis in oral biofilms. The discovery of such a dynamic would provide new key insights into biofilm physiology.

## Methods

### Enrichment of human salivary MUC5B and LDP for flow cell surface coating

Non-stimulated whole saliva from eight healthy individuals was collected on ice and pooled. MUC5B was isolated by isopycnic density gradient centrifugation as described previously [12,18]. Briefly, the pooled saliva was diluted 1:2 in 0.2 M NaCl and gently stirred overnight for solubilization. The sample was then centrifuged at 4,400 × g, 4°C for 30 minutes (Beckman Coulter Avanti J-E centrifuge, JA 20 rotor) to remove large particles and debris. The starting density of the supernatant was then set to 1.45 g/ml with CsCl before ultracentrifugation at 36,000 rpm, 15°C for 96 h (Beckman Coulter Optima LE-80 K Ultracentrifuge, 50.2 Ti rotor). Thereafter, 24 fractions of 1.7 ml were collected from the top of each tube and pooled per fraction. Fractions enriched with low density salivary proteins (LDP), were collected from the same material. Antibodies for the central domain of the MUC5B polypeptide backbone (6F10-E4, Novus Biological) and lysozyme as a representative LDP (EC 3.2.1.17) were used for ELISA to detect the fractions enriched with the proteins of interest. The fractions enriched with MUC5B or LDP were pooled separately to produce MUC5B- or LDP-enriched solutions, respectively, and were dialyzed against 10 mM phosphate buffer with 0.07 mM NaCl (PBS) (Spectra/PorTM Dialysis Membrane Biotech CE tubing, MWCO: 100 kDa for MUC5B or 3.5 kDa for LDP). Solutions were stored at −80°C until use. Protein concentrations were 0.3 mg/ml for the MUC5B-solution and 0.8 mg/ml for the LDP-solution, as determined by freeze drying and weighing after dialysis against water for salts removal.

### SDS-PAGE of LDP solution

**Figure 1.**
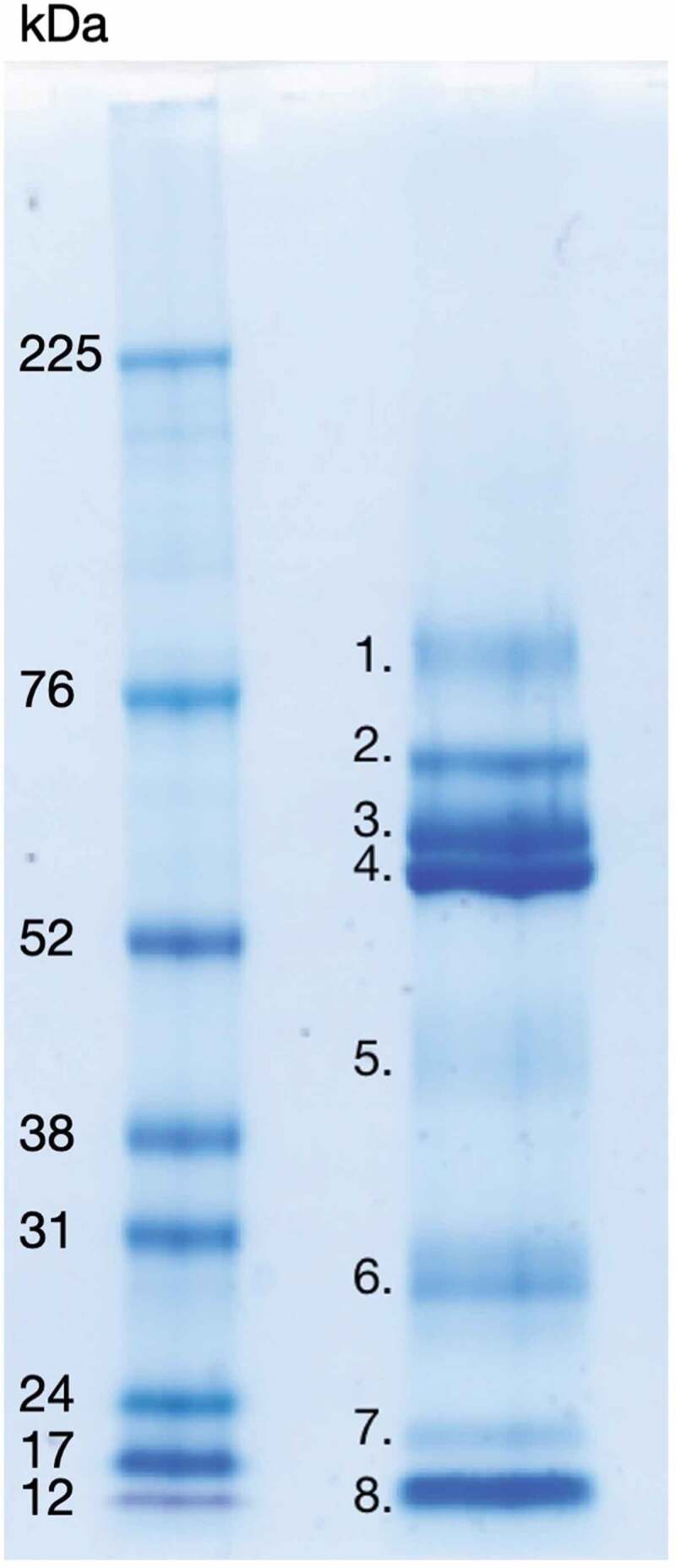
1D SDS-PAGE of LDP solution (right well), visualized with Coomassie Brilliant Blue stain. Protein bands visible with the Coomassie stain were identified by LC-MS/MS and are listed below. Left well, High-range Rainbow molecular weight marker (bio-rad). 1. Transmembrane secretory component. 2. Serum albumin pre-proprotein, Ig alpha-1 chain C region and alpha-amylase 1 precursor. 3. Alpha-amylase 1 precursor, unnamed protein product with molecular weight 54,127 Da, hypothetical protein with molecular weight 53,228 Da, immunoglobulin alpha heavy chain variable region (partial) and immunoglobulin G heavy chain variable region (partial). 4. Alpha-amylase 1 precursor, Ig alpha-1 chain C region, immunoglobulin alpha-2 heavy chain and bactericidal/permeability-increasing protein 1. 5. Carbonic anhydrase 6 isoform 1 precursor, unnamed protein product with molecular weight 38,775 Da, zinc-alpha-2-glycoprotein precursor, alpha-1-antitrypsin precursor, kallikrein-1 preproprotein, alpha-amylase 1 precursor and protein LEG1 homolog precursor. 6. IgG kappa chain (partial), immunoglobulin light chain (partial), unnamed protein product with molecular weight 18,867 Da, immunoglobulin variable region (partial), immunoglobulin lambda light chain VLJ region (partial), cystatin-SN precursor, unnamed protein product with molecular weight 66,151 Da and parotid secretory protein. 7. Prolactin-inducible protein precursor, unnamed protein product with molecular weight 18,867 Da, cystatin-SN precursor and putative lipocalin 1-like protein 1. 8. Prolactin-inducible protein precursor, cystatin-SN precursor, cystatin-S precursor, cystatin D, cystatin-C precursor, cystatin-SA precursor, thioredoxin isoform 1, cystatin-B and cystatin-A

To examine the protein composition, the LDP solution was subjected to 1D SDS-PAGE (Figure 1). Twelve μg (15 μl) LDP solution was added to 6 µl lithium dodecyl sulphate sample buffer and 2 µl reducing agent (Invitrogen), heated for 10 min at 70°C, spun down and loaded onto NuPAGE 4–12% Bis-Tris (Invitrogen) gels. Five µl undiluted high range rainbow molecular weight marker, 12–225 kDa, was used as reference standard in an adjacent well (Amersham, GE Healthcare). Gels were run with 3-(N-morpholino) propanesulfonic acid (MOPS) running buffer at 200 V for approximately 50–60 minutes at room temperature. Gels were then stained with Coomassie Brilliant Blue (Sigma) staining solution (16% Coomassie, 64% UHQ, 20% ethanol v/v) overnight, destained for 1 h in destaining solution as recommended by the manufacturer (25% ethanol in UHQ) and scanned at 600 dpi (MP Navigator EX 1.0 scanner). Visible bands were cut from the gels and identified with LC-MS/MS (Aberdeen Proteomics, University of Aberdeen) as described in a previous study from our laboratory [24]. LC-MS/MS data for Figure 1 can be found in the supplemental material.

### Bacterial strain and planktonic culture conditions for LC-MS/MS

*S. gordonii* DL1 was routinely grown overnight in 25% Todd-Hewitt Yeast Extract (¼ THYE, Becton Dickinson), at 37°C in 5% CO_2_. Cell cultures were diluted 1:10 in 25% THYE + 20 mM glucose (¼ THYE+G) and grown as described above until the mid-exponential phase (OD_600nm_ = 0.5–0.6) was reached. Planktonic cells were retrieved by centrifugation (3,000 rpm, 10 min, 5°C, 50 ml tubes, Beckman GS-6 R centrifuge), washed and resuspended in a 10 mM Tris HCl-extraction buffer pH 6.8, containing 1 mM ethylenediaminetetraacetic acid (EDTA) and 5 mM MgSO_4_, and stored at −20°C until protein extraction.

### Flow cell system for biofilm growth and harvest of biofilm cells for LC-MS/MS

The flow cell system used in this study is similar to a model previously described [23], but with larger dimensions. To produce the flow cell channels, two parallel glass slides were separated by two 1.6-mm rubber spacers mounted in a holder, sealed with rubber gaskets, and covered by a Perspex lid. Prior to each experiment, the glass slides were washed and sterilized as detailed in the previous study. Each flow cell had a total volume of 6.2 cm^3^, and a total surface area of 2 × 39 cm^2^ for biofilm growth. The flow was laminar, controlled by a peristaltic pump. The flow was 0.8 ml/min (shear flow 0.06 cm/s) during the adhesion and growth phases and 3.5 ml/min (shear flow 0.28 cm/s) during rinsing.

Solutions for surface conditioning were produced by adding MUC5B- or LDP-enriched solutions 1:2 to artificial saliva buffer pH 6.8 containing 15.6 mM KCl, 2.6 mM KH_2_PO_4_, 0.2 mM MgCl_2_, 2.6 mM Na_2_HPO_4_, 10 mM NaCl, 4.4 mM NH_4_Cl, modified from Björklund et al. [25] with CaCl_2_ (final concentration 0.1% w/v). The flow cell surfaces were pre-coated with the respective conditioning film at 37°C for 3 hours. Before inoculation, the flow cell channels were rinsed with ¼ THYE+G for 10 min.

*S. gordonii* DL1 cells were grown to mid-exponential phase and centrifuged as described for the planktonic cultures. Cell pellets were re-suspended in ¼ THYE+G to concentrate the cells tenfold. The cell suspension was then re-circulated over the flow cell surfaces for 2 h at 48 ml/h to allow the cells to adhere. After the adhesion phase, non-adherent cells were removed by rinsing with ¼ THYE+G for 1 h. The flow was then reduced and the biofilms were allowed to grow for 18 h with a continuous flow of ¼ THYE+G. Using a sharp blade, the adherent cell layers were then harvested into sterile PBS and thereafter handled as described for planktonic cells in preparation for protein extraction.

### Protein extraction for LC-MS/MS

Harvested cells, planktonic or biofilm, were subjected to three freeze-thaw cycles in the extraction buffer, centrifuged (3,000 rpm, 10 min, 5°C, 15 ml tubes, Beckman GS-6 R centrifuge) and resuspended in 700 μl lysis buffer containing 8 M urea, 2% (v/v) 3-[(3-cholamidopropyl)dimethylammonio]-1-propanesulfonate (CHAPS), 64.8 mM dithiothreitol (DTT), 2% immobilized pH gradient (IPG) buffer pH 4–7 (Pharmacia Amersham Biotech, Sweden). The suspensions were then ultrasonicated with homogenizing 0.2 mm glass beads for 4 × 5 minutes (5 second pulses and pauses, amplitude 40, Vibra-Cell^TM^ Ultrasonic Processor, SONICS), with alternate periods of cooling on ice. Intact cells and cell wall fragments were removed by centrifugation at 17,000 × g for 10 min at 4°C and the recovered supernatants (cellular protein extracts) were stored at −20°C until further processing. The protein concentration was determined using the 2-D Quant kit (GE Healthcare Life Sciences).

### Sample preparation for LC-MS/MS

For each replicate 60 μg protein was processed, precipitated, and subjected to proteolysis on hydrophilic interaction liquid chromatography (HILIC) microparticles (ReSyn Biosciences, Gauteng, South Africa) in a King-Fisher Flex (Thermo Fisher Scientific, Bremen). In 96-well plates, magnetic microspheres (1:10 protein:beads ratio) were incubated in equilibration buffer (15% acetonitrile (ACN), 100 mM NH_4_Ac, pH = 4.5), protein samples were incubated in binding buffer (30% ACN, 200 mM NH_4_Ac, pH = 4.5) for binding of proteins to HILIC beads, beads were washed twice in 95% ACN. Proteins were then digested for 1 h at 37°C with trypsin (20:1 protein:trypsin ratio) dissolved in 50 mM ammonium bicarbonate buffer, pH 8. Peptides were recovered from the plate and dried in a Speedvac (Thermo Fisher Scientific, Germany) prior to C_18_ desalting. Peptide desalting was performed using BioPureSPN Mini, PROTO 300 C_18_ (The Nest Group, Inc., MA, USA). Briefly, columns were equilibrated with 100 μl 70% ACN, 5% formic acid (FA) and conditioned using 100 μl 5% FA. Peptide samples were resuspended in 100 μl 5% FA and loaded on the column. Columns were washed with 5% FA before elution of peptides using 100 μl 50% ACN, 5% FA. The resulting peptide solution was dried by vacuum centrifugation and stored at −20°C until analysis.

### LC-MS/MS

Samples were resuspended in 10 μl 0.1% FA, and a volume corresponding to 300 ng was loaded onto an EASY-nano liquid chromatography (LC) system (Thermo Fisher Scientific, Germany). The analytical column was a silica capillary (75 μm × 16 cm Pico Tip Emitter, New Objective, USA) packed in house with C_18_ ReproSil-Pur 1.9 μm (Dr. Maisch GmbH, Germany). Peptides were separated using a 60 min LC gradient from 5% to 25% solvent B (80% ACN, 0.1% FA) and continuously sampled by a Q-Exactive HF-X Mass Spectrometer (Thermo Fisher Scientific, Germany) through an electrospray interface. Data were acquired using data-dependent acquisition (DDA) in positive ion mode. Precursor spectra (375 to 1,500 m/z) were acquired at 120,000 resolution with automatic gain control (AGC, MS1 target 3 × 10^6^) and a maximum injection time of 50 ms. The 20 most abundant ion peptides were continuously selected for fragmentation. Fragmentation spectra were acquired at 15,000 resolution with an AGC target of 1 × 10^5^ ions and a maximum injection time of 20 ms. Isolation width for fragmentation was set to 1.2 m/z.

### Mass spectrometry data processing and analysis

All raw mass spectrometry files were analyzed using MaxQuant (v 1.6.15.0) [26] applying the default settings with a false discovery rate (FDR) of 1% at both the peptide spectrum match (PSM) and protein level. Searches were performed against a database holding the *S. gordonii* proteome downloaded from UniProt [27] (uniprot-proteome_UP000001131). Intensity values for label-free quantitation (LFQ) [28] were processed using NormalyzerDE [26] and LFQ intensities normalized applying the CycLoess approach was used in subsequent analysis. All downstream analyses were performed using Perseus (v1.6.5.0) [29], a software package tailored for analysis of multidimensional omics data. For the interpretation of uniquely expressed proteins, only proteins with consistent presence or absence in all triplicates of each growth condition were considered. For protein abundance analysis, the data were first filtered for proteins detected in two or more replicates in at least one experimental condition. Missing values were then imputed from the low end of the proteome abundance distribution using the default setting in Perseus [30]. Proteins with differential abundance in uncoated, MUC5B and LDP coated conditions were identified through analysis of variance (ANOVA) (p < 0.05). Proteins categorized as differentially expressed in ANOVA were z-scored and visualized through hierarchical clustering using Euclidean distance and Pearson’s correlation for row and column tree, respectively. A fold change of >2 was set as the cut-off value for significant difference in abundance.

### S. gordonii DL1 and S. mutans UA159 adhesion to MUC5B

To compare the attachment to surface-bound salivary MUC5B of an early colonizing commensal to a later colonizing, more dysbiosis-related oral streptococcal species, *S. gordonii* DL1 and *S. mutans* UA159 were grown overnight in ¼ THYE (Becton Dickinson), at 37°C in 5% CO_2_. The separate cultures were diluted 1:5 in ¼ THYE+G and grown as described above until the mid-exponential phase (OD_600nm_ = 0.5–0.6) was reached. Cells were then centrifuged (3,000 rpm, 10 min, 5°C, 50 ml tubes, Beckman GS-6 R centrifuge) and resuspended in fresh ¼ THYE+G. Ibidi VI IbiTreat flow cell channels (Ibidi GmbH, Munich, Germany) had been pre-coated the night before with MUC5B- or LDP-conditioning solutions with CaCl_2_ (final concentration 0.1% w/v) and incubated at room temperature overnight. Biofilms were established by re-circulating the cultures over the pre-coated flow cell channels for 2 h at 0.06 ml/min (shear flow 0.06 cm/s) to allow the cells to adhere. After the adhesion phase, non-adherent cells were removed by gentle rinsing with sterile PBS, pH 7.5. Attached biofilm cells were stained using the BacLight™ LIVE/DEAD® viability kit (Invitrogen, Carlsbad, CA). Imaging was performed at 60 × magnification using a Nikon Eclipse TE2000 inverted confocal scanning laser microscope (CSLM) (Nikon Corp., Tokyo, Japan). An argon laser (488 nm laser excitation) fitted with a long-pass 515/30 filter for the green fluorescence signal and a long-pass 605/75 filter for the red fluorescence signal was used for illumination. Ten random images from each flow cell channel were captured and the experiment was performed in triplicates with independent bacterial cultures. Image analysis was performed using the software package BioImage_L [31] by calculating the % coverage in each image. Statistical analysis was performed using one-way ANOVA with a significance level of p < 0.05.

## Results

The objective of this study was to compare the cellular proteomes of an oral commensal, *S. gordonii* DL1, cultured planktonically to exponential growth phase or in biofilm flow cell systems for 18 hours. The biofilms were grown on three different surfaces, uncoated glass and glass surfaces coated with salivary MUC5B- or LDP-enriched conditioning films. All cultures were grown in a diluted complex medium (¼ THYE+G). Intracellular proteins were extracted and subjected to liquid chromatography–tandem mass spectrometry (LC-MS/MS) to study the cellular proteomes. Intensity values for all identified proteins can be found in Supplemental Table S1 (original) and 2 (with imputed missing values and statistical analysis).

### Uniquely expressed proteins in planktonic versus biofilm cells

**Figure 2.**
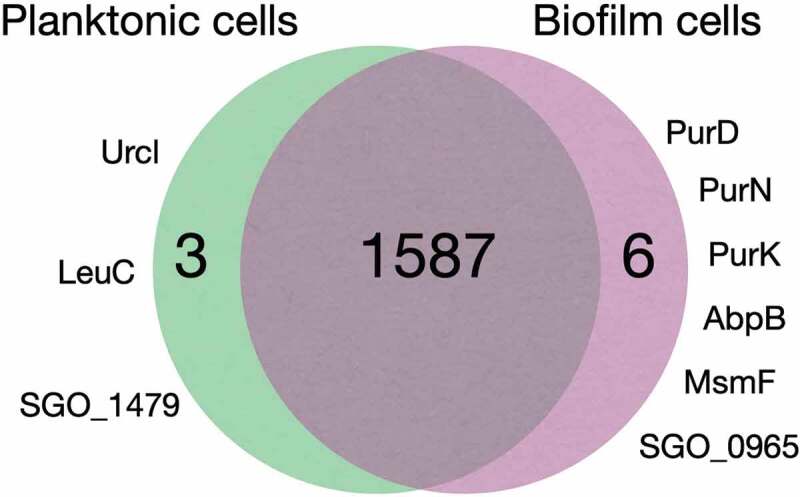
Comparison of proteomes in planktonic and biofilm cells (grown on uncoated and MUC5B- or LDP-coated surface). **Urcl**: Urease cluster protein, **LeuC**: 3-isopropylmalate dehydratase (large subunit), **SGO_1479**: Uncharacterized protein, **PurD**: Phosphoribosylamine–glycine ligase, **PurN**: Phosphoribosylglycinamide formyltransferase, **PurK**: N5-carboxyaminoimidazole ribonucleotide synthase, **AbpB**: Membrane dipeptidase (amylase binding protein), **MsmF**: Multiple sugar metabolism transmembrane permease, **SGO_0965**: Uncharacterized protein

1,587 out of the 1,597 identified proteins were shared by all cell cultures regardless of planktonic or biofilm growth or surface coatings. Six proteins, PurD, PurN, PurK, AbpB, MsmF and SGO_0965, were present in all biofilm cells but absent from the planktonic cells, and three proteins, Urcl, LeuC and SGO_1479, were absent from all biofilm cells, regardless of surface substrate, but expressed in planktonic cells (Figure 2 and Table 1). The β-glucoside operon antiterminator LicT was uniquely absent in the biofilm cells grown on MUC5B and present in all other cultures. Cellular functions associated with these proteins are summarized in Table 1.

**Table 1.** Cellular functions associated with uniquely expressed proteins in planktonic and biofilm cells

Novel biofilm cell proteins, absent in planktonic cells			
UniProt Acc No	Protein name	Abbreviation	Protein function
A8AZY0	Phosphoribosylamine–glycine ligase	PurD	Nucleotide metabolism; de novo purine biosynthesis.
A8AUA0	Phosphoribosylglycinamide formyltransferase	PurN	Nucleotide metabolism; de novo purine biosynthesis.
A8AZX7	N5-carboxyaminoimidazole ribonucleotide synthase	PurK	Nucleotide metabolism; de novo purine biosynthesis.
A8AUM3	Membrane dipeptidase (amylase binding protein)	AbpB	Proteolysis.
A8AXS5	Multiple sugar metabolism transmembrane permease F	MsmF	Transmembrane transport permease protein involved in multiple sugar metabolism (msm) mono- and disaccharide transport.
A8AWU6	Uncharacterized protein	SGO_0965	Suggested to be functionally associated with pdxK (SGO_0964) in *S.* *gordonii* DL1 based on gene neighborhood [26].
Planktonic cell proteins, absent in all biofilm cells			
A8AUP5	Urease cluster protein	Urcl	Urease assembly and activation.
A8AWP5	3-isopropylmalate dehydratase, large subunit	LeuC	Amino acid biosynthesis; Branched amino acid biosynthesis; L-leucine biosynthesis.
A8AY98	Uncharacterized protein	SGO_1479	Suggested to be functionally associated with a GNAT family acetyltransferase (sgo_1481) in *S.* *gordonii* DL1 based on gene neighborhood [26].
Proteins uniquely absent in MUC5B biofilm cells			
A8AXH7	β-glucoside operon antiterminator, BglG family	LicT	Inhibition of β-glucoside catabolite repression.

### Intracellular protein abundance profiles

**Figure 3.**
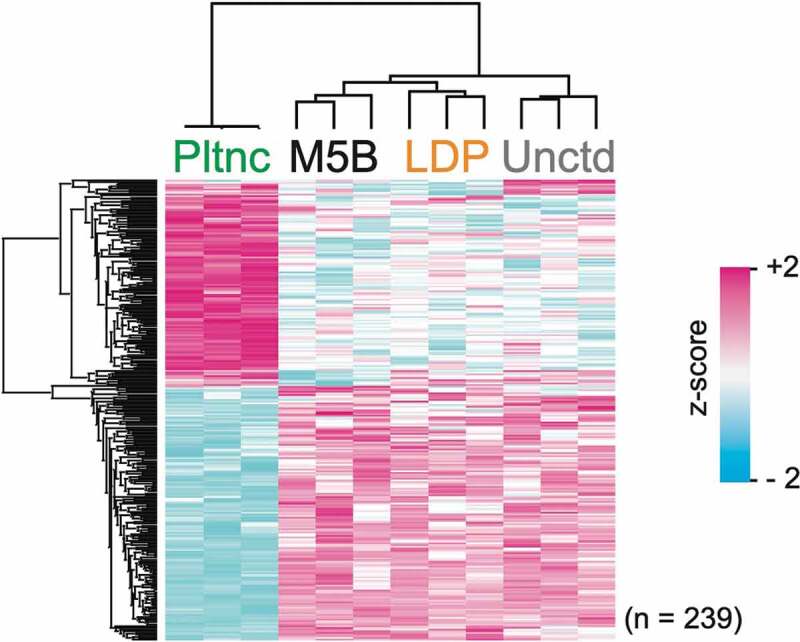
Clustering analysis. Hierarchical cluster of z-scored LFQ intensities for proteins with significant difference in abundance (ANOVA, p < 0.05; Benjamini–Hochberg FDR) between the different growth conditions

Overall, the protein abundance profiles differed the most for the planktonic cultures (Figure 3). As seen in Figure 3, between the three biofilm types, the protein abundance profiles of the biofilm cells grown on uncoated glass differed the most from those grown on the two salivary protein coatings.

#### Comparison of intracellular protein abundance profiles in biofilm cells grown on salivary MUC5B- and LDP-coated surfaces

**Figure 4.**
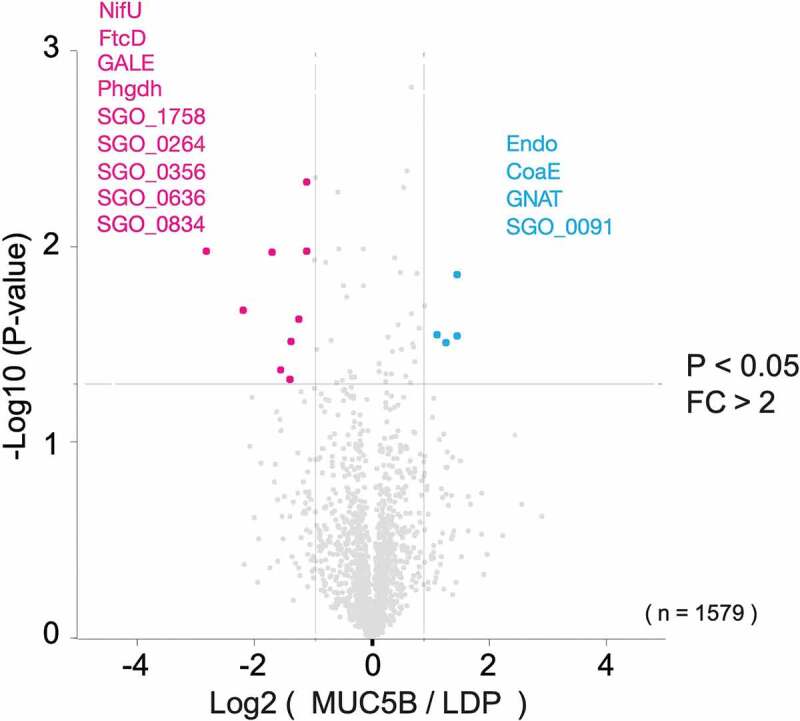
Volcano plot representation of proteins in *S. gordonii* DL1 biofilm cells grown on salivary MUC5B-coated surface versus LDP-coated surface from LC-MS/MS data. The lines indicate arbitrary cut-off values for enrichment (fold change > 2) and significance (p < 0.05). Proteins with significant difference in abundance are indicated in the figure and listed in Table 2

The protein abundance profiles differed in *S. gordonii* DL1 biofilms grown on salivary MUC5B compared to LDP (Figure 4). With a fold change of 2 as the cut-off for significant difference in expression, four proteins, Endo, GNAT, CoaE and SGO_0091, were identified as more abundant, and nine proteins, NifU, FtcD, GALE, Phgdh, SGO_1758, SGO_0264, SGO_0356, SGO_0636 and SGO_0834, were less abundant in biofilms on MUC5B, compared to biofilms on LDP (p < 0.05). Cellular functions associated with these proteins are summarized in Table 2.

**Table 2.** Protein abundances in MUC5B biofilms compared to LDP biofilms (p < 0.05, FC > 2)

Proteins more abundant in MUC5B biofilm cells compared to LDP biofilm cells				
UniProt Acc No	Protein name	Abbreviation	Protein function	Fold change
A8AUR8	Mannosyl-glycoprotein endo-beta-N-acetylglucosaminidase	Endo	Hydrolysis of high-mannose glycopeptides and glycoproteins containing the -(Man(GlcNAc) [2])Asn-structure. Putative role in immune evasion.	2.7
A8AVM2	GCN5-related N-acetyltransferase (GNAT family)	GNAT	Protein regulation. Putative role in immune evasion.	2.2
A8AW57	Dephospho-CoA kinase	CoaE	Coenzyme A biosynthesis.	2.7
A8AUF3	Uncharacterized protein	SGO_0091	Putative ABC transporter, suggested to be functionally associated with transcriptional regulator tetracycline repressor (TetR) family (SGO_0090) in *S.* *gordonii* DL1 based on gene neighborhood and gene co-occurrence [26].	2.5
Proteins less abundant in MUC5B biofilm cells compared to LDP biofilm cells				
A8AUC8	D-3-phosphoglycerate dehydrogenase, putative	Phgdh	Amino acid biosynthesis, L-serine biosynthesis.	−2.6
A8AYY1	SUF system FeS assembly protein, NifU family	NifU	Iron-sulfur cluster assembly.	−2.9
A8AZ18	Putative transcriptional regulator	SGO_1758	HTH-type transcriptional regulator.	−7.1
A8AZ65	Glutamate formimidoyltransferase	FtcD	Amino acid metabolism; Histidine catabolic process to glutamate and formamide.	−6.0
A8AZR0	UDP-glucose 4-epimerase BH3715	GALE	Carbohydrate metabolism; Galactose catabolism.	−3.0
A8AUX3	Uncharacterized protein	SGO_0264	Suggested to be functionally associated with Nac (SGO_0265) in *S.* *gordonii* DL1 based on gene neighborhood [26].	−2.2
A8AV65	Uncharacterized protein	SGO_0356	Suggested to be functionally associated with DegV (SGO_0357) in *S.* *gordonii* DL1 based on gene neighborhood and co-expression [26].	−2.6
A8AVX9	Uncharacterized protein	SGO_0636	-	−2.1
A8AWH3	Uncharacterized protein	SGO_0834	Suggested to be functionally associated with Nrd (SGO_0835) and PepV (SGO_0836) in *S.* *gordonii* DL1 based on gene neighborhood [26].	−3.2

### Comparison of S. gordonii DL1 and S. mutans UA159 adhesion to MUC5B

**Figure 5.**
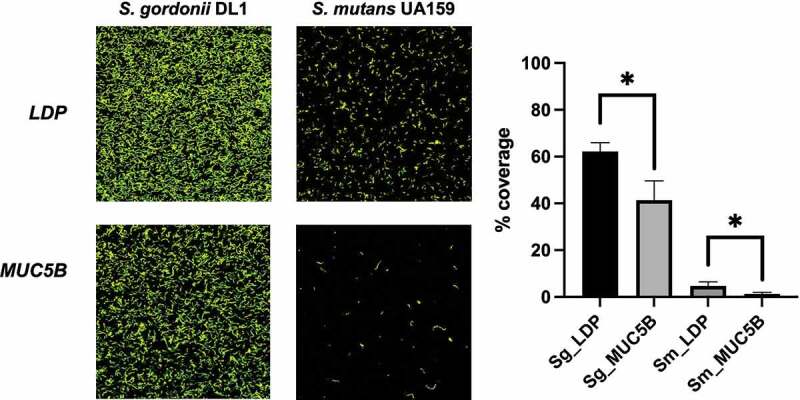
*S. gordonii* DL1 and *S. mutans* UA159 biofilms on LDP- and MUC5B-coated Ibidi VI flow cell IbiTreat surfaces after 2 h of flow. Differences in % coverage were calculated between all four groups using one-way ANOVA, * = p < 0.05. The experiment was performed in triplicate using independent bacterial cultures

The attachment to surface bound salivary MUC5B of the early colonizing oral commensal *S. gordonii* DL1, compared to the later colonizing, more dysbiosis-related *S. mutans* UA159 was studied in Ibidi VI flow cell systems using CLSM and fluorescent staining of biofilms (Figure 5). Surface bound MUC5B significantly reduced attachment of biofilms in both species compared to LDP-coated surfaces (p < 0.05), however, the relative reduction in attachment was greater in *S. mutans* UA159 (73.5%) compared to *S. gordonii* DL1 (33.5%).

## Discussion

Overall, the proteomic profiles of *S. gordonii* DL1 were similar across conditions, and 1,587 out of the 1,597 identified proteins were shared by all cell cultures regardless of planktonic or biofilm growth or surface coating. This can be compared to the total 2,058 proteins that are annotated for *S. gordonii* DL1 in the UniProt database [27]. The different growth conditions tested in this study were planktonic and flow cell biofilms on uncoated glass, and glass coated with salivary MUC5B or LDP. The aim of the study was to explore the effects of planktonic compared to biofilm growth, and of attachment to specific surface substrates, on the cell proteomes. It is worth noting that mass spectrometry of total bacterial cultures provides an average proteomic profile only. To detect differences in protein expression between subpopulations, alternative methodologies would be necessary. However, the differences in proteomic profiles across conditions that could nevertheless be detected underline the potential significance of these discrepancies and may inspire further investigation.

In accordance with previous findings [13], the planktonic protein abundance profiles deviated the most in relation to the biofilms. Comparing the three biofilm types, the protein abundance profiles of the biofilm cells grown on uncoated glass deviated the most from those grown on the two salivary protein coatings (Figure 3). The diverging effects of the uncoated glass surface on the biofilm cell proteomes from that of the physiological surface coatings underline the importance of utilizing authentic biological conditioning films in biofilm models to achieve a closer resemblance to the environment in the oral cavity. Furthermore, employing flow cell modelling, as opposed to static modelling, is an additional step towards better reproducing *in vivo* conditions.

Six novel biofilm cell proteins common to all biofilm cultures but absent in planktonic culture (PurD, PurN, PurK, AbpB, MsmF and one uncharacterized protein, SGO_0965) were identified. PurD, PurN and PurK are all involved in the *de novo* purine nucleobase biosynthesis pathway (PBP) [27]. PurE (UniProt accession number A8AZX8) and PurF (A8AU98), involved in the same pathway, were present in all biofilm culture triplicates but absent from two of the three planktonic triplicates. The remaining five member proteins of this pathway (PurA-C, PurH, PurM, UniProt accession numbers A8AZM9, A8AZX3, A8AU96, A8AUA2 and A8AU99) were present in all cultures with no significant difference in abundance. Purine is essential for all living organisms due to its role in a variety of cellular functions [32], and as demonstrated in *Streptococcus agalactiae* [33], growth is inhibited if the bacteria cannot either *de novo* synthesize or salvage purine from the environment. Four proteins were annotated with the GO terms for the biological processes related to purine salvage (GO:0043101, GO:0043096, GO:0006166 or GO:0032261) for *S. gordonii* DL1 in UniProt; adenylate kinase (Adk, A8AZK4), xanthine phosphoribosyltransferase (Xpt, A8AXD5), hypoxanthine phosphoribosyltransferase (Hpt, A8AZZ9) and adenine phosphoribosyltransferase (Apt, A8AWY0) [27]. All these purine salvage proteins were present in planktonic and biofilm cultures without any significant differences in abundance. The presence of the PBP Pur-proteins as well as purine salvage pathway proteins in biofilm cultures suggests that cells in this growth condition perform both *de novo* purine biosynthesis as well as employ salvage pathways for scavenging purine from the environment, while the absence of many of the Pur-proteins in planktonic cells suggest that suspended cells may primarily rely on salvage pathways.

Another protein identified only in the biofilm cultures was the membrane dipeptidase AbpB. This proteolytic protein is a hydrolase that acts as an aminopeptidase by cleaving single amino acids off the substrate protein N-terminal [27]. The AbpB polypeptide contains a signal peptide that is cleaved off before the protein is exported [34]. In *S. gordonii*, AbpB interacts with salivary amylase together with the amylase-binding protein AbpA. Based on sequence analysis, AbpA seems to be unique to *S. gordonii*, while AbpB shares sequence homology with other bacterial dipeptidases [34]. Both AbpA and AbpB seem to be involved in attachment during *S. gordonii* biofilm formation [35], however, related to its proteolytic activity, it has been suggested that AbpB also has a role in the acquisition of amino acids from environmental proteins [34].

Also, among the novel biofilm cell proteins was the multiple-sugar metabolism transmembrane permease F (MsmF), a transmembrane helical protein [27]. Based on reduced uptake and fermentation of a variety of carbohydrates in an MsmF-mutant, *S. mutans* MsmF was confirmed to be involved in the low-affinity multiple-sugar metabolism (msm) [36]. Several mono- and disaccharides have been identified as substrates for the msm system in *S. mutans* [37]. A phosphotransferase system (PTS) deficient mutant of *S. mutans* was found to switch to msm, which in turn reduced the total carbohydrate transport and metabolism in the cells [38]. A shift towards the low-affinity msm system may benefit biofilm lifestyle cells by reducing the flow through the central carbon metabolism and thereby protecting the biofilm against harmful acidification.

Urease cluster protein (here abbreviated Urcl), LeuC and one uncharacterized protein, SGO_1479 were absent in all biofilm cells and only identified in planktonic cells. Bacterial ureases in the oral cavity counteract acidification by converting urea from the saliva to ammonia and carbon dioxide [39]. In most bacteria, the urease enzyme consists of three subunits (UreA-UreC), with the assembly and catalytic activation also requiring up to six accessory proteins (UreD-UreI) [40]. Variants of this system are ubiquitous in many bacteria [40] and have been detected in oral streptococci [41], but have not been annotated in UniProt for *S. gordonii*, and Urcl is not a homologue to any of the urease cluster proteins UreA-I in other species [27]. Another protein absent from all biofilm cells that may also contribute to alkalinization through ammonia production [42] was LeuC. This protein is part of a heterodimer enzyme that consists of a large (LeuC) and a small subunit (LeuD) and acts as a lyase involved in biosynthesis of the branched chain amino acid (BCAA) L-leucine, together with LeuA and LeuB [27]. BCAA biosynthesis represents a cellular housekeeping process for amino acid metabolism, but the redirection of the carbon flow in the cell towards biosynthetic activities, and thereby reduced production of acidic end products, together with the production of NH_3_ are also used by *S. mutans* for protection against harmful local acidification [42]. The genes *LeuA-D* were assigned as acidurity genes after having been found to be significantly downregulated in a less aciduric mutant of *S. mutans* compared to the wild type [43]. While LeuC was absent from all biofilm cultures, LeuA-B and D were present in all cell types with no significant difference in abundance. This indicates that the pathway is present in both growth conditions, but is possibly downregulated or redirected in the biofilm cells. The dental biofilms depend on intricate molecular networks [44], and the expression of Urcl and LeuC in *S. gordonii* planktonic cells but absence of these proteins in biofilm cells suggests that this species may rather rely on other members in a mixed species biofilm, such as urease producing *Streptococcus salivarius* [41] and *Actinomyces* species [39,45,46], for local alkalinization by ammonia production from the urease or lyase pathways.

Surface-bound salivary MUC5B was found to provoke specific responses in the biofilm proteome. Four proteins (Endo, GNAT, CoaE and SGO_0091) were more abundant in biofilms grown on MUC5B compared to LDP (Table 2 and Figure 4). Endo is an N-glycosidase that hydrolyses high-mannose glycoproteins containing the -(Man(GlcNAc) [2])Asn-structure [27]. Bacterial N-glycosidases are suggested to be involved in cellular housekeeping functions, such as remodeling of macromolecules [47], for example, in cell wall recycling, but also in the extracellular release of saccharide moieties from human host glycoproteins to be utilized for nutrition. Secreted N-glycosidases, including N-acetyl-beta-D-glucosaminidase, from *Streptococcus oralis* were found to sequentially cleave off N-linked monosaccharides from human alpha1-acid serum glycoprotein, which in turn promoted bacterial growth [48]. Accordingly, deletion of endo-N-acetylglucosaminidase D in *S. gordonii* DL1 cultured in human whole saliva significantly reduced bacterial growth [49]. N-endoglycosidases are also involved in infection by assisting tissue colonization and invasion [50] and increasing virulence [51] in *Streptococcus pneumoniae*, and in immune evading functions by hydrolysis of human IgG in *S. pneumoniae* [52] and *Streptococcus pyogenes* [53,54]. Higher abundance of Endo in *S. gordonii* DL1 MUC5B biofilm cells indicates an enhanced ability to utilize N-linked glycoproteins as a nutrient source and may also facilitate bacterial colonization and putative immune evasion. This would benefit biofilm growth *in vivo* where saliva is the main nutrient source [2] and may increase species competitiveness through supporting colonization and evasion of the host immune system.

CoaE was more abundant in biofilms grown on MUC5B compared to LDP. This enzyme is a kinase involved in coenzyme A biosynthesis by catalyzing the phosphorylation of the 3ʹ-hydroxyl group of dephosphocoenzyme A to form coenzyme A [27]. In *S. mutans*, a dephospho-CoA kinase encoded by the *coaE* gene was reported as essential for bacterial growth [55]. The N-acetyltransferase GNAT was also more abundant on MUC5B compared to LDP, and enzymes belonging to this group catalyze acetylation of protein N-termini through the transfer of an acetyl group from acetyl-CoA to a variety of different substrates and are suggested to be involved in the regulation of carbohydrate metabolism, cell wall biosynthesis, antibiotic resistance, and other cellular functions in bacteria [56,57]. In *Streptococcus suis* serotype 2, a GNAT N-acetyltransferase family encoding gene was found to be involved in immune evasion from the host by enhancing phagocytic resistance in the bacteria [58].

One uncharacterized protein, SGO_0091, was more abundant in biofilms grown on MUC5B compared to LDP. This helical, transmembrane protein was found to be a putative ATP-binding cassette (ABC) transporter [27] and is suggested by gene neighborhood and gene co-occurrence to be functionally associated with a transcriptional regulator of the tetracycline repressor (TetR) family (SGO_0090) in *S. gordonii* DL1 [27]. In total, 12 TetR family transcriptional regulators are annotated in *S. gordonii* DL1 [27]. The products of the *tet* genes that are controlled by these transcriptional regulators confer resistance to tetracycline, hence, the name, but are also involved in a number of other biological processes, such as biofilm formation, catabolic pathways, stress responses, multidrug resistance and pathogenicity in both Gram-positive and Gram-negative bacteria [59].

The proteins that were less abundant in biofilms grown on MUC5B compared to LDP were NifU, FtcD, GALE, Phgdh, putative transcriptional regulator SGO_1758, and four uncharacterized proteins, SGO_0264, SGO_0356, SGO_0636 and SGO_0834. GALE is an isomerase that catalyzes the reaction UDP-galactose → UDP-glucose, the final step of the Leloir galactose metabolism pathway. UDP-glucose is then cycled back to the third step of the pathway, where it is converted into glucose-1-phosphate, the first intermediary of glycolysis, and then shunted into the central carbon metabolism [27]. GALE is necessary for continued Leloir pathway cycling [60]. In *S. gordonii* and *Streptococcus sanguinis*, Leloir pathway UDP-glucose is also utilized in the biosynthesis of cell surface presented polysaccharide coaggregation receptors [61]. The lower abundance of GALE suggests that UDP-galactose may be less utilized in the biofilms on MUC5B, leading to attenuated acid production and thereby a reduced risk for harmful acidification of the biofilm, and possibly also that different types of attachment strategies than the cell surface receptors produced from Leloir pathway UDP-glucose could be preferred in biofilms on MUC5B.

The putative transcriptional regulator SGO_1758 was also less abundant in biofilms on MUC5B compared to LDP. Based on gene neighborhood, this protein is suggested to be functionally associated with the isomerizing glutamine–fructose-6-phosphate aminotransferase (SGO_1757, GlmS) and glycosyl hydrolase 6-phospho-beta-glucosidase (SGO_1759, Ghg) [27]. GlmS is involved in the direction of carbohydrates from glycolysis to cell wall peptidoglycan synthesis through conversion of fructose-6-phosphate to GlcN-6-phosphate (GlcNAc precursor) in other bacteria [62]. The directions of the putative transcriptional regulations of SGO_1758 on GlmS and Ghg in *S. gordonii* are not known, however, it is interesting to note that Ghg is a product of the *bgl* operon; the same operon that is regulated by the transcription factor LicT that was uniquely absent in MUC5B biofilm cells (section 3.1).

The β-glucoside operon antiterminator LicT (UniProt accession number A8AXH7) was absent in the biofilm cells grown on MUC5B and present in all other cultures. LicT is a transcription factor belonging to the BglG family of transcriptional antiterminators, and it is present in a large variety of prokaryotes [27]. In the closely related *S. mutans*, the *bgl* regulon contains a set of genes that encode for a β-glucoside PTS carbohydrate uptake system [63]. β-glucosides include arbutin, salicin, cellobiose and aesculin, and represent secondary carbohydrates that are likely to be subject to catabolite repression in the presence of glucose [64]. LicT inhibits catabolite repression of β-glucosides and was found to enable continued transport and hydrolysis and thereby utilization of aesculin as a carbon source in *S. mutans* even in the presence of glucose, by maintaining expression of the β-glucoside PTS-specific Enzyme II and downstream metabolic enzymes expressed from the genes of the *bgl* regulon [63,65,66]. The ability to utilize secondary carbon sources even in the presence of glucose would possibly increase the acidity of the bacteria by providing access to a wider range of carbohydrates. *bgl* products were also associated with virulence factors in *S. pyogenes* related to soft tissue infection, such as blood dissemination and haemolysis [67], and in pneumococcal survival and virulence [68]. As opposed to in the other studied growth conditions, LicT was uniquely absent in *S. gordonii* DL1 biofilm cells grown on MUC5B. The absence of this transcription antiterminator results in continued repression of β-glucoside PTS-specific Enzyme II, and thereby reduction of acidity through reduced secondary carbohydrate uptake, and possibly also reduced expression of other putative *bgl* operon-related virulence factor proteins in the biofilm bacteria that bind to MUC5B.

In total, six novel biofilm cell proteins common for all biofilm cultures regardless of surface substrate and absent in planktonic culture (PurD, PurN, PurK, AbpB, MsmF and SGO_0965), and three proteins absent in all biofilm cultures, but expressed in planktonic cultures (Urcl, LeuC and SGO_1479) were identified in *S. gordonii* DL1. These differences may provide insights into mechanisms for adaptation to the biofilm lifestyle in this species. Moreover, the proteins shared between planktonic and biofilm cells are also interesting because they demonstrate potential core proteomes that may be consistent between growth conditions, potentially also between bacterial species. It is of interest to identify core proteomes because it enables subsequent identification of deviations from such core patterns. Such deviations may represent candidates in the development of biomarker tools or novel treatment strategies in relation to biofilm-induced disease.

Among the proteins that were upregulated in the *S. gordonii* DL1 biofilms grown on MUC5B compared to LDP, both Endo and GNAT have putative roles in carbohydrate metabolism and the evasion of the host immune system in other streptococci, and CoaE and SGO_0091 are tentatively associated with biofilm growth and other activities that would increase the competitiveness of a strain in a mixed biofilm. GALE and SGO_1758, both downregulated in biofilms on MUC5B compared to LDP, are suggested to regulate carbohydrate uptake and metabolism. Moreover, in the MUC5B biofilm cells, the transcription factor LicT, suggested to regulate carbohydrate uptake and thereby biofilm acidity through β-glucoside PTS-specific Enzyme II repression, was uniquely absent. While surface-bound MUC5B reduced attachment of biofilms in both *S. gordonii* DL1 and the later colonizer *S. mutans* UA159 compared to LDP-coated surfaces, the relative reduction in attachment was significantly smaller in *S. gordonii* compared to that in *S. mutans* (section 3.4, Figure 5). Based on these findings, it could be hypothesized that the early colonizing commensal *S. gordonii* which adheres well to surface-bound salivary MUC5B is favored by the specific proteomic responses elicited by this mucin, since many of these regulations seem to promote colonization and competitiveness of this species in dental biofilms. In the current study, analysis of proteomes presented in different experimental conditions on protein level was employed, as this is the direct determinant of phenotype. Indeed, gene activity may also be assessed at the transcript level. Future studies would benefit from employing a range of methodologies aimed at studying the roles of different salivary components in the modulation of biofilm subpopulation composition and activity over time, as well as including clinical isolates of a variety of species in mono- and multispecies cultures.

## Supplementary Material

Supplemental Material

## Supplemental online material

Supplemental Tables S1 and S2 and LC-MS/MS data for LDP SDS-PAGE are available from [link].
